# Nutrition and Specific Diseases in Women during the Life Course

**DOI:** 10.3390/nu15153401

**Published:** 2023-07-31

**Authors:** Nataliya Makarova, Birgit-Christiane Zyriax

**Affiliations:** 1 German Center for Cardiovascular Research (DZHK), Partner Site Hamburg/Kiel/Lübeck, 20246 Hamburg, Germany; 2 Preventive Medicine and Nutrition, Midwifery Science—Health Services Research and Prevention, Institute for Health Services Research in Dermatology and Nursing (IVDP), University Medical Center Hamburg-Eppendorf (UKE), 20246 Hamburg, Germany

In Western countries, the prevalence rates of risk factors for premature mortality and early non-communicable diseases are growing due to the increasing prevalence of poor nutrition habits, increasing levels of stress, and sedentary lifestyles.

The life course approach, developed by Diana Kuh, Yoav Ben-Shlomo and colleagues, offers an integrative approach which guides research on health, human development, and ageing [1]. The translation of this life course approach to women’s health, the study of reproductive health, is indispensable. It comprises the investigation of factors across one’s life that influence the timing of menarche, fertility, pregnancy outcomes, gynaecological disorders, and age at menopause. It also recognises the important influence of reproductive health on non-communicable disease risks in later life [2]. This integrative approach considers the continuity of reproductive health and the inter-relationship between different biomarkers and risk factors [2]. Within women’s life courses, lifestyle plays an essential role throughout different phases, ranging from young age to pregnancy, menopause, and healthy aging. Factors such as pregnancy outcomes, long-term health, quality of life, and disease risk are consequently influenced by women’s nutrition, levels of stress, physical activity, and over-riding lifestyle choices.

Correlations between nutrition and many diseases have been observed for many years. However, the underlying mechanisms and the effect sizes are only partly known due to the often multifactorial disease processes. The link between lifestyle and the growing rates of different diseases (e.g., cervical, ovarian carcinoma, breast cancer, cardiovascular diseases, or diabetes mellitus type 2) needs to be investigated further. New scientific approaches are being used to try to relate individual biomarkers to dietary patterns and changes in the microbiome in order to make risk potentials visible earlier. However, what are the key players in nutrition or physical activity that dominate lifestyle and need to be highlighted in prevention programs? More population-based studies and especially RCTs investigating the association between dietary factors and the occurrence of diseases are needed, leading to the central question: How can we prevent multimorbidity and reduced life quality in elderly women?

Within this Special Issue on nutrition and women’s health, we share twelve evidence-based research papers with the scientific community.

Currently, the average number of views within this Special Issue is 1649.

The first contribution within this Special Issue performed an analysis of the literature generated over the past 20 years regarding risks of uterine fibroids’ occurrence and dietary factors [3].

One study investigated the role of a high-fat, high-fructose diet in mice [4], whereas the remainder of the studies in this Special Issue investigated humans. The results from this animal model study showed that high-fat and high-fructose feeding given around puberty may directly affect reproductive and metabolic symptoms [4].

Four other research papers investigated cardiovascular disease risk factors within the large, population-based, longitudinal Hamburg City Health Study (HCHS) [5,6], the Australian Longitudinal Study of Women’s Health (ALSWH) [7], and a cross-sectional study from Taiwan [8]. In addition to conventional modifiable risk factors such as overweight, hypertension, hyperlipidaemia, and smoking or the consumption of alcohol, nutrition-related risk factors were highlighted as being more important. These included the intake of dietary supplements as well as psychologic risk factors such as depressive symptoms and social behaviour, including personality traits, political preferences, and altruism. The understanding of mechanisms of action between these risk factors allows clinicians to provide appropriate and tailored interventions in and disease prevention and management for women. A greater adherence to dietary quality scores, such as the Mediterranean dietary score or the DASH (Dietary Approaches to Stop Hypertension) score, was associated with a lower risk for and lower severity of risk factors. However, scores based on the Mediterranean diet were developed specifically for the southern European population and thus show weak adherence to diet, for example, in the German population.

A total of four studies highlighted the relationship between biomarkers such as GDF-15 [9], let-7g-5p [10] and germline BRCA1/2 mutation carriers [11]; metalloestrogens, GSTP1, and SLC11A2 polymorphisms [12] and dietary patterns and outcomes.

Finally, in a secondary analysis of the HeLP-her randomised controlled trial, postpartum weight retention (PPWR) was examined in non-Australian-born culturally and linguistically diverse women compared with Australian-born women [7]. 

Specific diseases in women such as letrozole-induced polycystic ovarian syndrome [4], uterine fibroids [3], vasomotor symptoms [13], and endometrial cancer [12] were examined within this Special Issue.

From the life course perspective, the phases of prepuberty [4], pregnancy [14], and postpartum [7] as well as breastfeeding [10], middle-aged [5,6], and postmenopausal [8] women were investigated. In one study, older women were compared to younger women [9]. Two studies compared men with women [5,6].

Despite the important role of lifestyle and the influence of nutrition on the development and course of diseases, there is little research considering nutrition in the preconceptional phase, in new-borns, and in young girls. Questions on how nutrition in these phases influence the life courses of women remain unanswered.

Further research with additional nutrition-related short-screening tools needs to be introduced to support clinical management and health services to identify patients with unhealthy diets. Additionally, the development of such tools can offer a simple aid for patients to improve their eating habits in a self-effective way.
